# Onchodermatitis: Where Are We Now?

**DOI:** 10.3390/tropicalmed3030094

**Published:** 2018-09-01

**Authors:** Michele E. Murdoch

**Affiliations:** Department of Dermatology, West Herts Hospitals NHS Trust, Vicarage Road, Watford, Hertfordshire WD18 0HB, UK; michele.murdoch@nhs.net; Tel.: +44-1923-217139

**Keywords:** onchodermatitis, onchocercal skin disease, onchocerciasis, ivermectin

## Abstract

Onchocerciasis causes debilitating pruritus and rashes as well as visual impairment and blindness. Prior to control measures, eye disease was particularly prominent in savanna areas of sub-Saharan Africa whilst skin disease was more common across rainforest regions of tropical Africa. Mass drug distribution with ivermectin is changing the global scene of onchocerciasis. There has been successful progressive elimination in Central and Southern American countries and the World Health Organization has set a target for elimination in Africa of 2025. This literature review was conducted to examine progress regarding onchocercal skin disease. PubMed searches were performed using keywords ‘onchocerciasis’, ‘onchodermatitis’ and ‘onchocercal skin disease’ over the past eight years. Articles in English, or with an English abstract, were assessed for relevance, including any pertinent references within the articles. Recent progress in awareness of, understanding and treatment of onchocercal skin disease is reviewed with particular emphasis on publications within the past five years. The global burden of onchodermatitis is progressively reducing and is no longer seen in children in many formerly endemic foci.

## 1. Introduction 

Onchocerciasis, caused by infection with the filarial worm *Onchocerca volvulus*, is one of the eleven neglected tropical diseases (NTDs) recently targeted for elimination by the World Health Organization (WHO) [1]. More than 99% of all cases are concentrated in 28 countries in sub-Saharan Africa. Small foci also occurred in the Americas, but there has been successful progressive elimination in this region and infection is currently found in a single large transmission zone (the ‘Yanomani area’) which straddles the border of Venezuela and Brazil [2]. Small foci of infection also persist in Yemen [3].

Historically onchocerciasis was better known for its clinical effects of visual impairment and blindness, prompting its alternative name of ‘river blindness’. Over recent years, however, there has been significantly increased awareness of the skin manifestations associated with this disease and indeed the main clinical manifestations of onchocerciasis in the twenty countries formerly covered by the African Program for Onchocerciasis Control (APOC) were related to skin disease [4].

Currently the WHO estimates that 198 million people are at risk of infection, though this number may increase as the mapping of areas of low transmission is finalized [5]. The Global Burden of Disease (GBD) Study 2013 estimated a global prevalence of 17 million infected cases [6]. The Democratic Republic of Congo (DRC) had the highest number of onchocerciasis cases at 8.3 million [7]. In its 2015 iteration, the GBD collaborators estimated an overall prevalence of 15.53 million, comprising 12.22 million with skin disease and 1.03 million cases with vision loss due to onchocerciasis [8]. The most recent available data in GBD Study 2016 estimates a global prevalence of 14.65 million [9].

When ivermectin was first licensed for human use in 1987 Merck, Sharp and Dome (MSD), now known as Merck and MSD, made the unprecedented decision to donate the drug (Mectizan^®^) to the world to treat onchocerciasis for as long as needed and it has remained the mainstay of treatment to date. In 2015 Dr. William Campbell, MSD and Prof. Satosh Ōmura of the Kitasato Institute shared the Nobel Prize in Physiology or Medicine for their development and use of ivermectin for onchocerciasis [10].

APOC was launched in 1995 with the objective of removing onchocerciasis as a public health and socio-economic problem in Africa [11]. The countries included in the program were: Angola, Burundi, Cameroon, Central African Republic, Chad, Congo, Democratic Republic of Congo (DRC), Equatorial Guinea, Ethiopia, Gabon, Kenya, Liberia, Malawi, Mozambique, Nigeria, Rwanda, South Sudan, Sudan, Uganda, and Tanzania. In 1997, APOC adopted community-directed treatment with ivermectin (CDTi) as its core strategy and the coverage and compliance with ivermectin steadily increased. In 2009 APOC changed its strategy to a target of elimination of the disease in Africa [12]. APOC closed at the end of 2015 and WHO established a new structure, the Expanded Special Project for Elimination of Neglected Tropical Diseases (ESPEN), to co-ordinate technical support for activities focused on five neglected tropical diseases in Africa, including onchocerciasis elimination [13].

Onchocerciasis control and elimination efforts are among the most sustained, successful, and cost-effective public health campaigns ever launched. By improving the general health of individuals, they contribute to improvements in worker productivity, gender equality and education and hence they actively contribute towards achieving several of the Millennium Development Goals [14].

## 2. Cutaneous Features

In 1989 Hay et al. reported an association between infection and skin changes associated with onchocerciasis in Ecuador [15]. The development of a formal clinical classification and grading system describing the cutaneous changes in onchocerciasis [16] facilitated more formal and extensive mapping of the true global burden of onchocercal skin disease (OSD). The categories of onchocercal skin disease delineated were (i) acute papular onchodermatitis (APOD) (ii) chronic papular onchodermatitis (CPOD) (iii) lichenified onchodermatitis (LOD) (iv) atrophy (v) depigmentation and (vi) hanging groin. The system was designed for easy use in the field by nurses or primary healthcare attendants, had good inter-observer variation *kappa* results and could be adapted for computer coding for large scale surveys.

A pre-control population survey of 6790 residents in savanna mesoendemic villages in Kaduna State, northern Nigeria [17] where onchocercal blindness was common, revealed that 38.6% of the residents aged five and above complained of itching with normal skin or had one or more forms of onchocercal skin disease including nodules. The presence of nodules was the most common finding (21.2%), followed by atrophy (6.1% of those <50 years), APOD (3.4%), depigmentation (3.2%), and CPOD (2.3%). A further 9.5% of residents complained of itching but had clinically normal skin. Atrophy, hanging groin and nodules were more common in females, whereas APOD was more common in males. After controlling for age and sex, microfilarial positivity was a risk factor for CPOD, depigmentation, hanging groin and nodules (OR 1.54, *p* = 0.046; OR 2.29, *p* = 0.002; OR 2.18, *p* = 0.002, and OR 3.80, *p* ≤ 0.001 respectively). Similar though weaker odds ratios were found with microfilarial load *per se*. 

The first multi-country study to explore OSD across Africa comprised seven rainforest or savanna-forest mosaic areas where onchocercal blindness was not common [18]. Following a census, individuals were randomly selected for examination in five of the study sites, though protocol deviation in the other two sites meant that individuals were asked to come to a central point for examination. Overall, onchocercal skin lesions (excluding nodules) affected 28% of the population aged five years and above. The commonest type of OSD was CPOD (13%), followed by depigmentation (10%) and APOD (7%). The prevalence of itching increased with age until 20 years and then plateaued, affecting 42% of the population aged 20 years and above. The prevalence of any onchocercal skin lesion and/or itching combined showed a very high correlation with the level of endemicity (as determined by the prevalence of nodules) of *r* = 0.8, *p* < 0.001).

In Yemen and Abu Hamid in Sudan, an atypical and severe form of onchodermatitis known as *sowda* (or lichenified onchodermatitis) is prominent. *Sowda* is common in older children, teenagers, and young adults but current expertise now suggests that all ages, including the elderly can be affected [19]. Typically, *sowda* presents as an extremely itchy hyperpigmented plaque or plaques on one leg; less commonly both legs or an arm or shouder can be involved. There is also often marked rubbery enlargement of the draining lymph nodes. Eye disease and palpable subcutaneous nodules are uncommon in Yemen and use of ivermectin has concentrated on treating skin disease in this country. A general concept is that onchocerciasis has a spectrum of skin changes, with *sowda* representing one end of a clinico-parasitological spectrum with low parasite loads and high levels of immune response.

A pre-control study in Edo State, Nigeria examined 2020 individuals who had visited primary health centers in each community and were recruited using simple random sampling. The area was hyperendemic for onchocerciasis with a skin snip positivity rate of 83%. The prevalence of depigmentation was very high at 87.5%, itching was 84.16% and nodules 75.42% [20]. Another pre-control study in Anfilo District of West Wellega, Ethiopia used a multistage sampling technique and a total of 1114 individuals ≥15 years were examined [21]. The prevalence of positive skin snips was 74.8% and nodules 12.1%. The prevalence of pruritus was 64.3%, leopard skin (19.1%), ‘skin lesions’ 11.3%, lymphadenopathy 16.4%, and hanging groin the least prevalent at 5.2%. The overall prevalence of pruritus and/or these clinical signs was 26.4%, being more prevalent in males (32.4%) than in females (20.8%, *p* < 0.05). A study in Enugu State, Nigeria revealed lichenified onchodermatitis was the most common clinical manifestation of onchocerciasis, occurring in 42/119 (35.29%) of infected persons (as denoted by the presence of palpable onchocercal nodules) [22].

There is a paucity of literature exploring concurrence of skin and eye morbidities. In a hyperendemic area of Cameroon, with a 63% nodule prevalence among males aged ≥ 20, individuals aged five years and older were invited to present themselves at a central point and 765 people were examined [23]. Onchocercal visual impairment (which included low vision and blindness) and depigmentation were found to concur significantly (OR 9.0, 95% CI 3.9–20.8), which was partly explained by age and exposure to infection (OR 3.0, 95% CI 1.2–7.7). Host immune characteristics such as the HLA-DQ alleles associated with depigmentation [24] might play a role in the pathogenesis of both depigmentation and visual impairment.

## 3. Imported Onchodermatitis

Growth in international travel and immigration means patients with onchocerciasis may be diagnosed in countries in the western world but it is probably under-reported because of its relatively non-specific presentations and limited awareness among physicians practicing outside endemic countries.

A retrospective study of 6168 patients diagnosed with one or more NTDs at a Tropical Medicine Referral Unit in Madrid, Spain between 1989–2007 found that onchocerciasis was the most common NTD in immigrants [25]. A diagnosis of definite onchocerciasis was based on positive skin snips or pruritus +/− skin lesions suggestive of onchocerciasis and a positive Mazzotti test (performed in patients with negative skin snips and no evidence of ocular onchocerciasis). Probable onchocerciasis was diagnosed in immigrants in the presence of pruritus +/− suggestive skin lesions and response to treatment with ivermectin. Onchocerciasis was present in 240 (9.1%) of immigrants (169 definite and 71 probable cases). All but two cases in immigrants occurred in African patients, with the majority coming from Equatorial Guinea (213/240, 88.8%), a reflection of the historical links between that country and Spain. The other countries of origin were Cameroon, Nigeria, Angola, and Zaire and one each from Republic of Guinea, Mali, Togo, D.R. Congo, Ghana, Sierra Leone, Sao Tome, Ivory Coast, Colombia, and Ecuador. (N.B. Zaire’s name was changed back to D.R. Congo in 1997). The number of new cases of onchocerciasis per new African immigrants significantly decreased each year over the period of the study. In a further group of immigrants who had travelled back to endemic countries to visit family and friends, there were 14 more cases of onchocerciasis.

With respect to the group of travelers who had visited endemic areas in this study, definite onchocerciasis was diagnosed in those with positive skin snips or positive serology in the presence of pruritus +/− suggestive skin lesions. In contrast to immigrants, who presumably had had long periods of exposure to infection prior to immigration, the number of travelers with onchocerciasis was much smaller at only 17. Of these, 16 had had a trip duration >3 months, range 3–336 months, and 1 patient had travelled for 1 month). All had travelled to sub-Saharan Africa and some patients had visited more than one country during their trip.

A literature search for English and French articles between 1994 and 2014 identified 29 cases of onchocerciasis in migrants from endemic countries and in expatriates and travelers from non-endemic areas [26]. The most frequent clinical manifestations in these cases plus the authors’ index case were pruritus (76.7%), unilateral leg or forearm swelling (43.3%) and rash (40%), whereas only two (6.9%) complained of eye symptoms. Eosinophilia was very common (92%). Eye symptoms, lymphadenopathy and chronic dermatitis were seen more frequently in migrants, whereas rash and arm swelling were more frequent in returned travelers and expatriates.

A review of 31 filarial cases in a French University Centre between 2002 and 2011 revealed 4 cases due to onchocerciasis comprising 3 immigrants from Cameroon, Sierra Leone, and Senegal with onchodermatitis and one traveler from Central Africa with arm swelling [27]. Another review of 289 NTD cases from 2000 and 2015 at the Infectious and Tropical Diseases Unit, Florence, Italy revealed just two cases of onchocerciasis from sub-Saharan Africa with typical cutaneous manifestations and they both presented within the first five years of the review [28].

In a group of 27 migrants who came to Israel from an onchocerciasis-endemic area in Ethiopia and who were referred for an atopic eczema-like rash, 14 had positive skin snips or positive IgG_4_ antifilarial serology [29]. The migrants who did not have laboratory proof of infection had similar clinical findings. Considering the group as a whole, patients’ main complaint was relentless pruritus, which began with an average of 2.2 years after immigration, which a range of 1 year prior to immigration to 11 years after immigration. The most common finding was LOD in combination with atrophy and depigmentation 8/27 (30%), followed by CPOD 7/27 (26%).

The largest case series of imported onchocerciasis to date reviewed 400 cases attending a reference clinical unit in Madrid, Spain [30]. All the migrants came from sub-Saharan countries and the most frequently occurring dermatological symptom was pruritus.

## 4. Burden of Disease

Onchocerciasis is mainly a non-fatal disease and its public health impact is therefore best understood in terms of DALYs (DALYs = Years of Lives Lost (YLL) + Years Lived with Disability (YLD)). Both skin and eye disease caused by onchocerciasis result in a decrease in productivity [31]. Initially only the burden from onchocercal eye disease was considered in global estimates, but the burden from ‘itch’ was first included in the GBD Study 1990, based on data from the multicountry prevalence study in Africa [18]. Physical onchocercal skin disease manifestations have also been included since the GDB Study 2010. There is now evidence that *O. volvulus* infection is causing onchocerciasis-associated epilepsy including nodding syndrome, and this condition is associated with high mortality, because in remote onchocerciasis-endemic regions a large number of individuals are not treated or treated too late with anti-epileptic drugs. Such sequelae have not been included in GBD estimates to date.

The GBD study 2013 estimated that onchocerciasis was the sixth highest cause of NTD-related YLDs globally and it was ranked highly in the top 10 leading causes of YLDs in Liberia, Cameroon and South Sudan. In all these countries, the burden from onchocerciasis is predominantly due to onchocercal skin disease [7]. In its 2015 iteration the GBD Study provided an overall global estimate of 1,135,700 (YLDs) due to onchocercal infection [8]. In the GBD Study 2016, onchocerciasis was ranked as the first leading cause of YLDs for Liberia, as the second leading cause for DRC and South Sudan, the fifth for Cameroon and the sixth cause for both Central African Republic and Sierra Leone [9].

## 5. Immunopathogenesis

Filarial parasites are known to induce a large range of immunoregulatory mechanisms to evade and down-modulate the host’s immune system in order to ensure the parasite’s survival [32]. Such mechanisms include induction of regulatory T cells, which promote high levels of non-complement binding IgG_4_ [33]. Survival of *O. volvulus* within the human host is thus the result of a complex interplay with the host’s immune system, which itself may be dependent on genetic factors, and pathology ensues when pro-inflammatory processes override any immunomodulatory effects.

*Wolbachia* are endosymbiotic bacteria found in most human filariae, (except *Loa loa)*, and appear to be essential for the filarial worm’s fertility and survival. In an experimental murine model *Wolbachia* were found to be an essential component in the development of anterior segment onchocercal eye disease and mediated corneal pathology by activating Toll-like receptors on mammalian cells, which in turn stimulated recruitment and activation of neutrophils and macrophages [34].

Recruitment of neutrophils by *Wolbachia* around adult female worms in *O. ochengi* infection in cattle has been shown to confound eosinophil degranulation and may act to protect the adult worms from the host immune system [35]. Furthermore, the major inflammatory motif of *Wolbachia* lipoproteins are able to directly activate human neutrophils in vitro [36].

The formation of neutrophil extracellular traps (NETS), a process referred to as NETosis, is now regarded as a novel effector mechanism, consisting of the extrusion of nuclear contents with neutrophil-derived granular and cytoplasmic proteins, which may limit microbial spread by entrapment and limit collateral inflammatory tissue damage by entrapping and degrading soluble cytokines and chemokines. Tamarozzi et al. visualized extracellular NETS and neutrophils around adult *O. volvulus* in nodules excised from untreated patients but not in nodules from patients treated with doxycycline, which kills *Wolbachia.* [37]. In addition, whole *Wolbachia* or latex microspheres coated with a synthetic *Wolbachia* lioprotein of the major nematode *Wolbachia* TLR2/6 ligand, peptidoglycan associated lipoprotein, induced NETosis in human neutrophils in vitro and TLR6-deficient mice were used to demonstrate that TLR6 was essential for this process. It is possible that NETosis triggered by *Wolbachia* is an anti-parasite response to limit the density of tens of thousands of uterine-released microfilariae (mf) produced daily by each adult female worm and that *Wolbachia*-induced NETs may directly modify inflammatory processes in the skin.

TGF-β was preferentially observed in the skin of infected individuals with ‘generalized’ or hyporeactive onchocerciasis and was reduced in patients with the hyperreactive form of onchocercal skin disease (LOD or *sowda*) [38]. In a similar vein, ‘hyperreactive onchocerciasis’ has been found to be characterized by a combination of accentuated Th17 and Th2 immune responses and reduced regulatory T cells [39].

Secretory extracellular superoxide dismutase (*Ov*ES-SOD) from *O. volvulus,* which is found in the excretory/secretory products of adult worms, was able to trigger responses in sera from onchocerciasis patients, with IgG titers significantly higher in sera from individuals with the ‘hyperreactive’ form compared with sera from those with the generalized form of onchocerciasis [40]. The authors proposed that, in addition to its role in superoxide anion reduction in the extracellular space, the *Ov*EC-SOD may help regulate inflammatory responses.

In patients who became mf-negative after repeated ivermectin treatments, parasite-specific cellular immune responsiveness and Th1 and Th2-type cytokine production becomes reactivated. Similarly, mf-negative patients after repeated ivermectin treatments have enhanced pro-inflammatory chemokines and reduced regulatory chemokines and cytokines [41].

Immunocytochemical examination of nodules using immuno-markers for blood and lymphatic vessels has suggested an intimate relationship between adult *O. volvulus* worms and lymphatic vessels, including the likely proliferation of lymphatic endothelial cells [42]. This has raised the possibility that the lymphatic system may be more involved in the migration of adult *O. volvulus* worms than was previously believed and may explain the lymphoedema that is sometimes seen in onchocerciasis [16]. Microfilariae, which have been documented in the blood in heavily-infected onchocerciasis patients and after treatment, might also migrate via the lymphatic system. Angiogenesis and lymphangiogenesis within nodules is characterized by the expression of CXCL 12, CXCR4, VEGF-C, angiopoietin-1 and angiopoietin-2. A proportion of macrophages in the inflammatory infiltrate in nodules were positive for the lymphatic endothelial cell marker Lyve-1 and some were integrated into the endothelium of the lymphatic vessels [43] and angiogenesis and lymphangiogenesis within nodules may provide new targets for drug treatment.

### Imported Skin Disease Pathogenesis

Baum et al. [29] noted a long interval for some Ethiopian immigrants in Israel before they developed any symptoms and hypothesized that environmental factors resulting from immigration from a developing to an industrialized country triggered an immunological shift to strong T-helper (Th) 2 responses, in a similar manner that an increased prevalence in asthma had been noted in Ethiopian migrants several years after migrating to Israel.

## 6. Immunogenetics

HLA class II variants may influence susceptibility to infection by *O. volvulus* and subsequent host immune responses causing pathology. Correlation between allelic variants of HLA-DQA1 and HLA-DQB1 and various forms of onchocercal skin disease have previously been documented in a Nigerian population [24], and recently a protective role of DQA1*0401 against *O. volvulus* infection has been demonstrated in both Cayapas Amerindians and Afro-Ecuadorians. Furthermore HLA-DQA1*0102 and *0103 seemed to represent risk factors for infection in Afro-Ecuadorians and HLA-DQA1*0301 was a possible susceptibility allele in the Cayapas population [44].

## 7. New Diagnostics

The quest for elimination of onchocerciasis requires newer, more sensitive diagnostic tests to verify that transmission of infection has been suppressed or interrupted. Such tests differ from previously used tests to diagnose infection in individuals.

### 7.1. Detection of Parasite in Humans

The sensitivity of the skin snip assay has been increased by replacing microscopic examination of the snip with detection of amplified parasite DNA. Most assays target the tandemly repeated sequence in the *O. volvulus* genome called the 0–150 repeat. Real-time PCR and isothermal loop amplification (LAMP) assays have also been developed [45,46]. On comparison of three PCR methods for evaluating onchocerciasis elimination efforts in areas co-endemic with other filarial nemaodes, the qPCR-O150 assay was deemed to be more appropriate for evaluating skin snips of OV-16 positive children when deciding when to stop MDA [47]. A novel O-5S qPCR assay targeting the *O. volvulus* O-5S rRNA gene, had 100% specificity and proved more sensitive than O-150 qPCR assay (66.5% vs 39% positivity rate) [48].

### 7.2. Serological Tests to Detect Exposure to O. volvulus

The Ov16 ELISA is now recommended by WHO guidelines for demonstrating the interruption of transmission of *O. volvulus* [49]. According to these guidelines, the serological threshold for stopping MDA is an Ov16 antibody prevalence of <0.1% among children under 10 years of age who act as sentinels for recent infection, but the current tools are not reliably specific enough and an Ov16 threshold of <2% may ultimately prove to be the most reliable serological threshold for stopping MDA [50]. Current assays have focused on IgG_4_ detection, but the IgG_4_ response takes time to develop and thus will not immediately reflect recent exposure. Two commercially available rapid diagnostic tests (RDTs) are a single Ov16 test and a combination test using Ov16 and the *W. bancrofti* antigen Wb123 [51]. The SD BIOLONE Ov16 rapid test was successfully field-tested in Senegal [52].

### 7.3. Detection of Parasite in Vector Blackflies

The O-150 PCR DNA amplification assay is the most widely used assay to screen pools of flies to verify elimination of transmission. Instead of using human bait to catch the vector blackflies, as has been done in the past, the Esperanza window trap has been used in Mexico with success [53,54] and such traps are being evaluated for use in Africa [55].

### 7.4. Detection of Biomarkers

Recent research has also produced assays to detect potentially viable adult worms such as specific metabolites produced by female worms [56,57] and detection of parasite microRNA in the blood [58,59] though the latter may not be present in sufficient concentration to act as a biomarker for infection [60].

## 8. Treatment

### 8.1. Effect of Ivermectin on Cutaneous Disease

Ivermectin, a macrocyclic lactone, interacts with post-synaptic glutamate-gated chloride channels resulting in paralysis of mf, which are therefore transported to regional lymph nodes and killed by effector cells. Release of uterine mf is also temporarily inhibited.

The first multi-country study on the short-term effect of ivermectin on onchocercal skin disease in Africa was performed by Brieger and colleagues in four study sites in Nigeria, Ghana and Uganda [61]. They followed up rural villagers for 18 months and found that from 6 months onwards, the prevalence of severe itching was reduced by 40–50% among those receiving ivermectin compared to the trend in the placebo group. The prevalence of APOD, CPOD, and LOD combined was significantly reduced in the ivermectin group at 9 months and the severity at 3 months. Furthermore, there was no difference between ivermectin given at 3, 6, or 12 monthly intervals.

The first assessment of the effect of mass treatment with ivermectin in the Onchocerciasis Elimination Program for the Americas was Banic et al.’s report [62] in the hyperendemic Yanomani communities of Roraima State, Brazil. Pre-treatment, 18/103 individuals (17.5%) had atrophy +/or ‘scaling’ of the skin. After three years of twice-yearly ivermectin therapy, there was a very significant reduction in the prevalence and intensity of infection by skin snips but there was no reduction in the prevalence of nodules or onchodermatitis.

The first multi-country study on the longer term impact of ivermectin on onchocercal skin disease involved seven study sites in Cameroon, Sudan, Nigeria and Uganda [63]. Two cross-sectional surveys were performed at baseline and after five or six years of CDTi. In phase I, 5193 individuals were examined and 5180 people participated in phase II. Within each study site, 10 villages underwent a census to cover approximately 1500 persons. Individuals aged five years and above were asked to present themselves for examination at a central point until a sample size of approximately 750 was obtained. The effect of five or six rounds of annual CDTi was profound with significant (*p* < 0.001) reductions in the odds of itching (OR 0.32), APOD (OR 0.28), CPOD (OR 0.34), depigmentation (OR 0.31) and nodules (OR 0.37). Reduction in the odds of LOD was also significant (OR 0.54, *p* < 0.03).

In Anfilo district, Western Ethiopia, 971 participants aged 15 years and above were examined after 6 years of annual CDTi and the prevalence of microfilaridermia, pruritus, leopard skin, nodules, and hanging groin were reduced by 45.6%, 54.4%, 61.3%, 77.7%, and 88.5% respectively [64].

In a previously hyperendemic rainforest area with a nodular rate of ≥40% in Anambra State, Nigeria, a cross-sectional survey of 894 subjects after a decade of CDTi identified nodules in 86 (9.62%) persons and 186 (20.81%) had one or more forms of onchocercal skin disease. There was a total absence of OSD in children < 10 years old and only 5 (5.43%) with OSD in the second decade of life, indicative of some encouraging success of the CDTi program. The rate of APOD however increased with age up to the third decade and decreased thereafter suggesting on-going transmission, either due to poor compliance or low coverage of treatment. All the individuals with APOD had missed the annual ivermectin treatment more than once during the program. CPOD, LOD, ATR, and DPM all increased with age for both sexes [65].

In 2015 after more than 15 years of CDTi in the West Region of Cameroon, a cross-sectional survey of 2058 individuals aged 5 years and above was performed to assess progress towards elimination. The weighted prevalence of positive skin snip results was 5.5% and that of nodules 2.1%. The weighted prevalence of skin disease excluding nodules was 1.7% and varied from 1.1% in men to 2.2% in women. Of note, treatment compliance was again found to be poor with only 39.3% of participants declaring they had taken five treatments during the last five years [66].

Prior to control measures on the island of Bioko, Equatorial Guinea, a survey in the mid 1980s reported that 28.8% of the study population suffered from dermatitis, pigmentation changes and cutaneous atrophy. After vector elimination in 2005 and more than 16 years of CDTi on Bioko Island, a community-based cross-sectional survey was performed in 2014, including a full cutaneous examination [67]. Although these workers found that 50.4% individuals reported never having taken ivermectin and only 28% had taken it more than twice within the past five years, there was a reduction in pruritus and skin lesions (14.9% complained of pruritus, 3% had nodules, 1.3% had ‘onchodermatitis’ and a further 1.8% had leopard skin. Nodules were more common in subjects older than 10 years and pruritus was more frequently found in adults (17.6%) than children (5.9%, *p* = 0.002).

With standard annual dosing, ivermectin was initially thought to have minimal macrofilarical activity, but recent mathematical modelling suggests that multiple doses of ivermectin, even at standard (150 µg/kg) doses and annual frequency, can have a modest permanent sterilizing effect after four or more consecutive treatments. The life expectancy of adult *O. volvulus* was reduced by 50% and 70% respectively after three years of annual or 3-monthly treatment with ivermectin [68]. There have been reports of suboptimal responses in some patients in Ghana after repeated treatment. In a Ghanian study of 42 patients treated with ivermectin and 204 randomly selected individuals, a significantly higher *MDR1* variant allele frequency was noted in suboptimal responders (21%) than in patients who responded to treatment (12%) or the random population sample (11%). CYP3A5*1/CYP3A5*1 and CYP3A5*1/CYP3A5*3 genotypes were also significantly different for responders and suboptimal responders, suggesting a possible role of these haplotypes in an individual’s response to ivermectin [69].

In Yemen, ivermectin was initially distributed only to *sowda*, (or lichenified onchodermatitis), cases four times a year, but a mass drug distribution program to treat the entire community has now begun. In the northern endemic valleys, there has been a marked reduction in the number of *sowda* cases from more than 50% pre-drug treatment to approximately 6% and in most areas it is uncommon to find new cases of *sowda* [19].

### 8.2. Effect on Imported Skin Disease

In Baum et al.’s study of Ethiopian immigrants to Israel [29], both patients with confirmed, and those with suspected, onchocerciasis, responded equally to ivermectin with reduction in itching and lichenification. Overall 9/17 (52%) had remission of more than 12 months, 5/17 (30%) had temporary relief lasting 3–12 months and required repeat treatment and 3/17 (18%) did not respond to treatment.

Puente’s case series [30] reported that ivermectin was used as first-line therapy and adverse events were described in 11 (3.2%) cases.

### 8.3. Effect of Ivermectin on Psychosocial and Socio-Economic Aspects of Onchodermatits

In the past sufferers with OSD were considered unclean and were stigmatized because of fear of transmission of OSD, resulting in social ostracism. OSD has also been associated with reduced productivity [31], difficulties breastfeeding, poor school attendance and reduced marriage prospects for affected teenage girls. Vlassof et al.’s pre-control multicountry study in Africa had identified that one third of residents with OSD reported low self-esteem, about half of those affected perceived onchocercal skin disease as a very serious health issue and 1–2% had even considered suicide [70]. Higher levels of stigma were noted in individuals with APOD, CPOD or LOD than persons with depigmentation.

After a decade of CDTi, a questionnaire presented to subjects in Anambra State, Nigeria identified that itching and onchocercal skin manifestations remained the most troublesome symptom and sign of onchocerciasis and social seclusion (or stigmatization) the most worrisome consequence. A preponderance of onchodermatitis on the limbs (visible area of the body to others), plus involvement of the buttocks (an area considered ‘private’) were deemed contributory factors for the psychological impact of the skin disease [71]. In a random sample exit interview of 594/40,914 persons treated with ivermectin in Ezinihitte, Nigeria, (an area with predominantly onchocercal skin disease) the most common reason cited for seeking treatment was ‘to gain treatment and prevention of skin problems’ [72]. The fifth and sixth rank-order reasons were ‘to prevent hanging groin’ and ‘to prevent/relieve enlargement of the scrotum or clitoris’. Genital lymphoedema is caused by filarial blockage of lymphatics in the pelvic region. Although both hanging groin and genital lymphoedema have low prevalence, they have important implications for married life and sexuality.

A multicountry study in Africa in Cameroon, DRC, Nigeria, and Uganda after at least four years of CDTi used random sampling of household treatment records to capture factors that reflected individuals’ perception of benefits of CDTi. In this study, overall 84.7% of respondents indicated that ivermectin treatment had many benefits: social benefits included improved ability to work, peer acceptance and improved school attendance; individual benefits included self-respect/esteem, election to political office and improved domestic relationships and health benefits included improved skin texture and less ill health. Improvement in skin was perceived for the individual by 40.4% and for the household by 39.5% of respondents. Reduction in itching was perceived for the individual by 54.5% of respondents and for the household by 52.2% of respondents [73].

A subsequent multicountry study using multi-stage sampling after 7–10 years of CDTi revealed that although people with OSD were still stigmatized and people still feared sexual intimacy with affected persons, avoidance of people with OSD had decreased from 32.7% before CDTi activities to 4.3% [74]. People who had lived in the community for less than 5 years tended to stigmatize those with OSD more than those who had lived in the community for longer and the youth stigmatized the most. Reasons given for avoiding people with OSD included ‘considered infectious’, ‘looked ugly’, ‘were irritating’, ‘were dirty’, ‘were scary’ and ‘were embarrassing’. An example of the changes in perception towards OSD is this quote from a young Nigerian man in a focus group discussion: *“Although we know better now, there is still the fear that something like hanging groin is hereditary. Really, people no longer avoid sufferers so much but I know that here we think that if it gets to the stage where one’s groin is hanging, then it will be hereditary. Before no-one would go into marriage with a girl whose mother had leopard skin because it was believed that she would develop it and no female would ordinarily marry a man whose father had hanging groin. But these things are changing now because we know better”.*

An interesting study asked schoolchildren aged 6–16 years to draw their perceptions of onchocerciasis and CDTi in their communities. Out of a total of 50 drawings generated, 30 pictures were categorized as showing symptoms of the disease, which included rashes and swellings (nodules), and a further 5 represented multiple perceptions on symptoms, benefits, and effects of treatment [75]. The results highlighted that children were cognizant of the external signs of onchocerciasis and the authors recommended that children be included in health promotion activities to maintain successful compliance with CDTi.

## 9. Update on Onchocerciasis Control Programs and Elimination

### 9.1. Onchocerciasis Elimination Program for the Americas (OEPA)

Right from its outset in 1993, this program used six-monthly mass ivermectin distribution with a target coverage of 85% with the goal of elimination of onchocerciasis from the region. Ivermectin distribution four times/year was also used in some areas. WHO has recently produced guidelines to help countries know when they can safely stop MDA and transition to a period of post-treatment surveillance (PTS) based on entomological evaluation to detect infection in the blackfly vector and serological evaluation in humans to detect the presence of antibodies to *O. volvulus* Ov16 antigen [49]. When all foci in a country have satisfactorily completed the PTS, the country may request a visit by a WHO verification team to assess elimination of transmission. By the end of 2012, transmission had been eliminated in 11 of the 13 foci in the Americas [6]. Elimination was first demonstrated by Colombia in 2103 [76], followed by Ecuador in 2014, Mexico in 2015 and Guatemala in 2016 [77]. Elimination of transmission in Ecuador was particularly gratifying as the main vector here, *Simulium exiguum*, was a very effective transmitter of infection and the skin and eye disease in this focus was probably the most severe in the Americas [78].

The remaining two onchocerciasis foci in the Americas form a single epidemiological transmission unit (the Yanomani Area) along the border between Brazil and Venezuela. There are challenges to treating the Yanomani Area, which is a remote area and difficult to reach. Furthermore, the Yanomani people can freely move across the country borders whereas program officials cannot, so increased political co-operation between the countries is needed.

### 9.2. African Program for Onchocerciasis Control (APOC)

The African Program for Onchocerciasis Control (APOC) initially focused on control of the disease as a public health problem. It was uncertain whether ivermectin could actually interrupt transmission and eliminate the parasite in Africa, as here the vectors are very efficient and the epidemiology very different, with large endemic areas that were often not well defined. The first evidence that elimination of onchocerciasis with ivermectin treatment was feasible in Africa came from studies in Senegal and Mali published in 2009 [79] and 2012 [80], which led to a paradigm shift from one of control of the disease to a target of elimination.

A further encouragement came from Tekle et al.’s report of a skin snip survey in 3703 individuals above the age of one year performed after 15–17 years of CDTi in Kaduna State, Nigeria. (These were the same villages where the onchocerciasis skin classification had originally been field-tested). These workers found that all examined individuals were skin snip negative, which was the first evidence from an APOC country that elimination of onchocerciasis infection with ivermectin might be feasible in Africa [81]. Unfortunately, Boko Haram activities interrupted fieldwork and entomological evaluations are still awaited.

From its outset APOC included a small number of projects where it was judged that local eradication of the vector would be possible and cost-effective and could be combined with CDTi. The island of Bioko, Equatorial Guinea [82], and the Itwara focus in Uganda [83] both achieved vector elimination. Bioko Island had no subsequent reported cases of infection and a recent study on 5–9-year-old school children revealed no evidence of infection by skin snipping and blood spot for Ov16 and Wb123 IgG_4_ [84]. Current WHO serological criteria for stopping MDA were therefore met and 3 years of post-treatment surveillance are currently underway to identify any new cases of infection.

The Abu Hamed focus in Sudan, which had predominantly the severe form of skin disease *sowda* or lichenified onchodermatitis, was the first focus in Africa to have successfully completed the entire WHO-recommended process to confirm elimination [85].

In 2007 Uganda launched a national elimination policy based on twice-yearly ivermectin treatment and vector control/elimination. By 2017, 1,157,303 people in six foci were living free of onchocerciasis, which is the largest population to date declared free under WHO elimination guidelines, providing further evidence that elimination of onchocerciasis in Africa is possible [86]. Ethiopia, Mali, Niger, and Senegal also have eliminated onchocerciasis in subnational areas. Although APOC faced certain challenges it achieved overall major success as a control program [11]. All areas where *O. volvulus* might be transmitted and where ivermectin has not been distributed in the past, now require careful ‘elimination mapping’ to determine whether they are onchocerciasis endemic or not so that appropriate treatment plans can be made [87].

### 9.3. Yemen

Since February 2016 Yemen has been using MDA with ivermectin in *sowda*-endemic areas with the goal of eliminating onchocerciasis in that country [88].

### 9.4. Challenges Faced by APOC

APOC faced several challenges, especially in conflict and post-conflict situations and in areas co-endemic with *Loa loa.* In DRC for example, the country had been devastated by political unrest and two civil wars and even after the signing of peace in 2003, fighting continued in the eastern provinces. Although the annual therapeutic and geographical coverage of CDTi projects slowly increased from 2001–2012, targets could not be met [89]. In Sierra Leone, civil conflict also resulted in limited onchocerciasis control activities from 1991–2002, but after the war, good CDTi was achieved between 2005 and 2009. In 2010, after 5 rounds of ivermectin, 10 out of 12 endemic districts had a >50% reduction in mf prevalence and 11 of 12 districts had ≥50% reduction in mean mf density among the positives, suggesting that Sierra Leone will now be on course to achieve elimination by the year 2025 [90].

Co-endemicity with *L. loa* has been another significant challenge for APOC. In areas co-endemic with loaisis, ivermectin treatment in people with high loads of *L. loa* mf can cause severe and occasionally fatal encephalopathy reactions. Little was known about the geographical distribution of loiasis in DRC at the start of CDTi projects and in 2004 adverse events in CDTi areas co-infected with loiasis resulted in 14 deaths [89]. Mass treatment was temporally halted whilst the situation was re-evaluated. A rapid assessment procedure for *L. loa* which assesses an individual’s history of eye worm (RAPLOA) was subsequently introduced in co-endemic areas. If ≥40% of the population report eye worm, this is deemed to pose an unacceptable risk of encephalopathy and MDA is withheld from that area. Recently the LoaScope, a mobile phone-based imaging device that can rapidly determine the mf density of *L. loa* infections, has proven useful in determining more accurately whether or not MDA can safely proceed. In the Okola health district of Cameroon, persons with very high *L. loa* microfilarial counts (>20,000 mf/mL) were thus able to be excluded from ivermectin therapy and no serious adverse events occurred [91]. Individuals at risk of ivermectin-related side-effects in loiasis- co-endemic areas may safely be treated with doxycycline but a course of treatment (4–6 weeks) is required.

Additional challenges to APOC included cross-border transmission of infection. Although Uganda has some areas clear of disease, conflict in the north of the country meant that maximum control activities have only been carried out over the past 3–4 years and cross-borders areas continue to cause difficulties because of delay in programs in the DRC and South Sudan. A strategy meeting between Uganda and DRC, initially triggered by the Ebola outbreak, led to improved cross-border co-operation for onchocerciasis control and elimination [92] and lessons learnt from the Sierra Leone, Liberia, and Guinea (Conakry) Mano River Union collaboration on onchocerciasis should help with other neglected tropical disease programs in the future [93]. Although international borders that intersect endemic regions present the biggest challenge, intra-country borders (e.g., administrative districts, or loiasis-endemic and non-endemic areas) can also pose problems [94]. Migrant populations are also part of cross-border challenges. Non-compliance with treatment has been another issue is some areas [95] but can be improved using traditional kinship structures [96]. Hostility towards health workers occurred during the Ebola outbreak as some people feared they were responsible for spreading Ebola [97].

### 9.5. Health and Economic Impacts of MDA with Ivermectin

Using updated disability weights for visual impairment, blindness, and troublesome itching (0.033, 0.195 and 0.108 respectively), Coffeng et al. estimated that APOC had cumulatively averted an impressive 19 million DALYs from 1995 up until 2015 [98]. This represented some 80% reduction in loss of DALYs for APOC countries, though in reality the true burden averted by APOC is even larger still as these updated estimates did not include disfiguring skin disease and onchocerciasis-associated epilepsy, including nodding and Nakalanga syndrome [99,100,101].

Redekop et al., considered mild and moderate skin disease and moderate and severe vision loss and blindness and estimated that the global economic benefit (productivity loss prevented) for the period 2011–2030 for onchocerciasis was 7.1 billion I$ (International dollars) if a target of elimination by 2020 was achieved [102].

GBD 2010 data has been used to estimate the global health impact of meeting the London Declaration 2012 targets on NTDs [103]. Regarding onchocerciasis, for the period 2011–2020, 7 million DALYs were averted and for 2021–2030, 12.6 million DALYS, giving a total of 19.6 million DALYS averted over the entire period, compared to a counterfactual scenario of no control/elimination program. The projected health benefits were thus deemed to justify the enormous effort involved. With respect to Ethiopia, the GBD study 2015 data, estimated that the age-standardized DALY rates for onchocerciasis have encouragingly decreased by a dramatic 66.2% between 1990 and 2015 [104].

Using a mathematical dynamical transmission model called ONCHOSIM, Kim et al. simulated trends for the prevalence of severe itching, low vision, and blindness in two scenarios of elimination of onchocerciasis in Africa versus a control scenario of continuing measures simply aimed at keeping the disease at a locally acceptable level [105]. Using the same vision disability weights as above but a disability weight for severe itching of 0.187 [106,107] Kim and colleagues estimated that elimination of the disease in Africa would avert 4.3 million–5.6 million (DALYs) over 2013–2045 compared with staying in the control mode. The decrease in the prevalence of severe itching was faster than those of low vision and blindness and the majority of DALYs averted were associated with the reduction in severe itching cases.

As ivermectin is a broad-spectrum anti-parasitic agent, it also has an effect on so-called off-target diseases, including soil-transmitted helminthiasis, lymphatic filariasis and scabies. Krotneva et al. [108] have estimated that between 1995 and 2010 annual MDA with ivermectin cumulatively averted about an extra 500 thousand DALYs from these co-endemic infections. This represents approximately an additional 5.5% relative to the total burden of 8.9 million DALYs averted from onchocerciasis, thus indicating that the overall cost-effectiveness of APOC is even higher than previously thought.

### 9.6. Effect on HIV

As HIV and helminth co-infection may be associated with a higher viral load and lower CD4+ cell counts, treatment with ivermectin could potentially benefit people living with HIV beyond simply treating the worm infection. Specific evidence for this to date is limited but there is no suggestion that anti-helminthic drugs are harmful for HIV-positive individuals [109]. NTDs may also lead to a worse prognosis in TB and malaria sufferers and further research on these interactions is needed [110].

### 9.7. ESPEN

Successful integrated chemotherapy for both onchocerciasis (with ivermectin) and lymphatic filariasis (ivermectin + albendazole) has been underway in some co-endemic areas [111]. APOC has now closed and a new programme has been established called the Expanded Special Project for Elimination of Neglected Tropical Diseases (ESPEN). This new program has different funding and governance and aims to co-implement control activities of onchocerciasis alongside other neglected tropical diseases and WHO currently recommends the use of preventive chemotherapy (PC) for lymphatic filariasis, onchocerciasis, schistosomiasis, soil-transmitted helminthiasis (hookworm, ascariasis and trichuriasis) and trachoma.

## 10. Newer Treatments

Alternative (or complementary) strategies (ATSs) are needed in some African settings in order to achieve elimination of onchocerciasis by 2025. Examples of ATSs include additional vector control [112,113] biannual or pluriannual CDTi, community-directed treatment with combinations of antihelminthics or new drugs and ‘test and treat’ strategies. As itching can reappear several months after an annual dose of ivermectin there should be more advocacy for bi-annual ivermectin distribution, but this is logistically difficult in remote areas.

### 10.1. Anti-Wolbachia Treatments

In loiasis co-endemic areas, treatment with ivermectin carries the risk of serious and sometimes fatal reactions due to the associated rapid killing of *L. loa mf.* As *L. loa* does not contain *Wolbachia*, anti-*Wolbachia* antibiotics such as doxycycline can be safely used to treat onchocerciasis in co-endemic areas. Antibiotics that are already registered for human use are undergoing evaluation for anti-*Wolbachia* activity to try to identify drugs that could have shorter treatment regimes, than the current six-week course of doxycycline. High-dose rifampicin has had promising results in animal studies [114]. A Cochrane review performed in 2015 [115] identified three randomized controlled trials that compared the effectiveness of doxycyline plus ivermectin versus ivermectin alone. The authors concluded that there was only limited evidence of very low quality from two of the studies that a six-week course of doxycyline followed by ivermectin may result in more frequent macrofilaricidal and microfilaricidal acitivity and sterilization of female adult worms compared with ivermectin alone. Only one study measured clinical outcomes, which were visual outcomes at six months but the results were graded as very low quality and hence the vision-related outcomes were uncertain. Similar RCTs assessing skin-related outcomes have not been reported to date.

In loiasis co-endemic areas, community-directed delivery of a six-week course of doxycycline proved feasible and doxycycline was a safe and effective macrofilaricidal agent [116]. A meta-analytical model using field trial data estimated that the efficacy of doxycycline (the maximum proportional reduction of adult female *O. volvulus* worms positive for *Wolbachia*) was 91–94%, irrespective of a variety of treatment regimes of four, five or six weeks. The life span of adult worms was reduced by 70–80%, from approximately 10 years to 2–3 years [117]. A pilot trial in Ghana confirmed that a four-week course of doxycycline was sufficient for *Wolbachia* depletion and that minocycline 200 mg/day for three weeks was more potent than a three-week course of doxycycline [118]. An Anti-*Wolbachia* Consortium (A-WOL) has been established to look for new drugs with macrofilaricidal activity by targeting *Wolbachia* and the capacity of this screening program has been significantly enhanced via the development of a high-throughput assay [119].

### 10.2. Moxidectin

Moxidectin is a more effective microfilaridial agent than ivermectin and 12 months after moxidectin treatment, dermal mf were still lower or comparable to the nadir seen one month after ivermectin treatment [120]. A double-blind, parallel group superiority trial in four study sites in Ghana, Liberia and DRC confirmed that at 12 months post-dosing, the skin microfilarial density was lower in the moxidectin group than the ivermectin group (adjusted geometric mean difference 3.9 [3.2–4.9], *p* < 0.0001) [121]. EpiOncho modelling suggests that the number of years to reach thresholds for onchoerciasis elimination with annual moxidectin is similar to that with biannual CDTi [122]. A not-for-profit organization, Medicines Development for Global Health, is planning for affordable access to moxidectin for countries to incorporate moxidectin into their control and elimination programmes. Moxidectin now has FDA approval for onchocerciasis in adults but further research is needed to clarify where and when it will be possible to use it on a community-wide basis.

### 10.3. Ivermectin-Diethylcarbamazine-Albendazole

Triple therapy is being considered, but a lot more work is still needed to identify whether or not it has a role in treatment of onchocerciasis. A strategy is needed to ensure that *O. volvulus*-infected patients with high microfilarial loads are excluded from treatment, as diethylcarbamazine can cause general and irreversible ocular side effects [123].

### 10.4. Emodepside

Emodepside, which has known efficacy in animal models, paralyses adult filarial worms by facilitating a nematode Ca^2+^-activated K^+^ channel called SLO-1 in a sustained way, but does not affect human channels [124]. It is therefore hoped it may prove to be a useful macrofilaricidal agent for human use and is undergoing a phase 1 study to determine its safety, tolerability, and pharmacokinetics in healthy volunteers by the Drugs for Neglected Diseases Initiative (DND*i*) (NCT02661178).

### 10.5. Genome Assemblies

Recently genome assemblies for *O. volvulus* and *Wolbachia* have been generated, allowing identification of enzymes that are likely to be essential for *O. volvulus* survival. This will hopefully provide a rich resource of potential new targets for drug development [125].

### 10.6. Vaccine

In 2015 an international consortium launched a new global initiative, known as TOVA (‘The Onchocerciasis Vaccine for Africa’), with the goal of evaluating and pursuing vaccine development as a complementary control tool to eliminate onchocerciasis. Two recombinant proteins, *Ov*-103 and *Ov*-RAL-2, have been identified that individually or in combination induced significant protection against infection in animal models [126], and it is hoped that initial vaccine candidates could be in human safety trials by 2022 [127].

## 11. Concept of Skin NTDs and Integrated Control and Management of Neglected Tropical Skin Diseases

In addition to onchocerciasis, several other neglected tropical diseases (NTDs) have cutaneous manifestations and a new proposal is for an integrated strategy for the management of skin NTDs using preventive chemotherapy, or intensified disease management, or both, depending on the overall health needs of an area [128]. Such an approach will require (i) assessment of which diseases are present within an area (ii) roll-out of training packages to help workers screen for several conditions and (iii) care pathways for diagnosis and treatment in the local community and onward referral to health centers and district hospitals as needed, with appropriate strengthening of health infrastructure. Targeting skin NTDs should also help treat other common skin conditions and hopefully lead to wider public health benefits. WHO has recently produced a training guide to help front-line health workers recognize NTDs through examination of the skin [129], and the key pointers identified for onchodermatitis were (i) itchy skin, (ii) subcutaneous lumps (large lumps suggest onchocercal nodules; small itchy lumps suggest acute or chronic onchodermatitis) and (iii) patches (raised dark scaly patches on one leg suggests lichenified onchodermatitis, and non-itchy speckled loss of pigment on shins suggests onchocercal depigmentation).

Hofstraat and Brakel reviewed social stigma towards NTDs in general and proposed that further research was needed to study the efficacy of joint approaches to reduce stigmatization in society and that lessons learnt from leprosy should be incorporated [130].

## 12. Mathematical Modelling of Onchodermatitis

The mathematical model ONCHOSIM has been extended to include predicted trends for various forms of onchocercal skin disease up to 2025 [131]. The prevalence of reversible skin disease (e.g., troublesome itching, acute and chronic papular and lichenified onchodermatitis) was shown to decline rapidly with waning infection prevalence, with the rate of the decline depending on achieved therapeutic coverage. In contrast, irreversible manifestations such as cutaneous atrophy, depigmentation and hanging groin declined much more slowly. ONCHOSIM has its drawbacks as it was not set up initially for chemotherapy. Other elimination simulation models exist, including SIMONA which has been used in the Americas.

## 13. Conclusions

In 2016, more than 131 million people were treated with ivermectin for onchocerciasis and 85.9% of all districts globally had achieved effective coverage of ≥65% [132]. As a result of MDA with ivermectin, onchocerciasis has been significantly reduced in many countries, transmission has been eliminated in four Central and South American countries and in foci in several African countries, and onchodermatitis is no longer seen in children in many formerly endemic foci. Continued vigilance is needed to check for the development of resistance to ivermectin, a single-dose macrofilaricidal agent remains the ‘Holy Grail’ for drug developers, and alternative strategies need to be implemented in some areas. Much concerted effort needs to continue to hope to achieve WHO’s target for elimination of onchocerciasis by 2025. Even after transmission has been interrupted, certain individuals will have irreversible deforming hanging groin, visible depigmentation and onchocerciasis-associated epilepsy but hopefully the incessant and debilitating pruritus due to onchocerciasis will become a thing of the past.
